# Different Deeds, Different Needs—Types of Violence Against Women and Social Support Sought Online

**DOI:** 10.3390/bs14111066

**Published:** 2024-11-07

**Authors:** Tinkara Pavšič Mrevlje, Vanja Ida Erčulj

**Affiliations:** Faculty of Criminal Justice and Security, University of Maribor, Kotnikova ulica 8, 1000 Ljubljana, Slovenia; tinkara.pavsic@um.si

**Keywords:** online support, support types, violence

## Abstract

Women, as victims of violence, among which intimate partner violence prevails, venture online to seek a supportive community. Members of online support groups differ in the experiences and needs they have and support they seek. The objective of this research was to explore the difference in types of support sought and support received between women who self-report having been a witness/victim of violence and others who directly ask for help without explaining their circumstances. For this purpose, content analysis of 600 randomly selected starting posts from an online support community was performed. The results reveal an association between the needs expressed and the experience of violence described. Although users most frequently sought informational support, those recounting sexual violence more often asked for emotional support or were looking for an emotional release. Posts describing a different kind of violence were more likely to bring more support than short posts directly asking for help. The findings are important since women in a violent relationship tend to become more isolated or controlled by their partners, pointing to the crucial role of online support in helping and encouraging those women to take the first step in seeking help from formal institutions.

## 1. Introduction

Violence against women includes any act of force that seriously threatens a woman’s life, body, psychological integrity, or freedom, where the most prevalent is the abuse of women by a former or current intimate male partner [1]. One in three women has been the victim of intimate partner violence (IPV) or another type of violence in their lifetime [2]. The classification of IPV includes physical, sexual, and/or psychological abuse [3]. Economic abuse was also recognized as a unique type of violence where the perpetrator controls the victim’s ability to acquire, use, and maintain the resources needed for everyday survival [4]. The most frequent type of violence is psychological violence [5]. It includes emotional abuse or controlling behavior and can accompany other types of violence. In the EU, the average prevalence of psychological violence is 43% with a large variation between countries [5].

Social support has been found to be an important factor in mitigating and moderating the consequences of IPV and improving health outcomes [6]. It is defined as the provision of assistance or comfort to others, typically to help them cope with different stressors [7]. Cutrona and Suhr [8] outline five types of social support. The most common types of support are informational and emotional. Informational support is providing knowledge or facts like advice or feedback on actions to the support seeker, whereas emotional support is the evaluation and acknowledgement of an individual’s feelings or providing comfort, empathy, sympathy, and encouragement. Three less common types of support are instrumental, esteem, and social network. Instrumental (tangible) support is providing any required goods and services to the support seeker, esteem support includes promoting one’s skills, abilities, and intrinsic value, and network support enhances one’s sense of belonging to a given group with a similar interest or sharing a similar experience or situation.

A study on a large representative sample in the U.S.A. showed that women in a continuing or recent physically abusive relationship experience more negative reactions (arguments and demands) from their relatives and friends, causing them even greater distress. Those who had experienced physical violence from their partner, but not in the last year, had stronger social support from their relatives and friends [9]. Research findings suggest that social support from relatives and friends, specifically emotional and instrumental, is a protective mechanism against IPV. Among the two sources of support, support from family members seems to be the most effective, resulting in less frequent and severe intimate partner violence [10]. Not only can social support act as a buffer in an abusive relationship, but it can accelerate changes in victims’ lifestyles, assisting them in finding a way out of such a relationship [11].

Social support can be formal or informal, online or offline. There are many advantages of online social support for female victims of violence. First, it can be easier to overcome the shame and guilt that might deter an abused woman from acting on the decision to leave a violent relationship or seek face-to-face help [12,13]. Second, the fact that online groups are easy to access and constantly present is an important aspect of the quality of online social support [14]. Moreover, members of online support groups may have similar experiences that can increase the understanding between support seekers and providers, and lead to social support that better matches the former’s needs. Another crucial element of social support quality is that social support has a greater effect when it meets the support seeker’s needs [15].

Nevertheless, the effectiveness of such support also depends on the way a victim seeks it. It can be direct, complete with explicit details, and directly asking for help (direct strategy), or indirect by vaguely complaining, dropping clues about an issue, or avoiding the topic (indirect strategy) [16]. It is understood that those seeking help directly receive better support, even though many other factors like shame, fear, guilt, etc., might prevent victims from being more direct [17].

When seeking help online on Q&A sites, women generally seek advice about financial, health, and legal issues, ask general questions about IPV, enquire about choosing one of the two possible options, or seek explanations concerning “the nature of the abusive relationship, the reasoning behind actions, the process for task completion, and the potential outcomes of events” [18]. Perhaps because conclusions are based on the analysis of Q&A sites, there is a larger extent of informational support seeking and provision among members, while emotional support comes in the form of encouragement. The prevalence of informational support in online support groups on IPV was also established in another study [19] in which the authors also found that women spent little time actively online and their contribution to the online discussions was small. On the other hand, a study in China showed that female victims of IPV mostly receive emotional support (e.g., offering encouragement and empathy), only then followed by informational support (providing explanations and giving information about immediate and long-term actions) [20]. Another study [21] revealed that victims of sexual violence primarily visited an online group to find a supportive community, seek advice, and share their own stories. Rich storytelling was found to be one of the techniques for obtaining emotional support [22]. Victims of sexual abuse more often need emotional support that validates their experience and offers clarification and labeling [23], which will in turn allow the victim to take further, more concrete steps. Other types of social support have rarely been identified in research studies analyzing the content of online discussions. Some women in a Chinese study sought instrumental support, like free temporary lodging, albeit such posts were rare [20].

A meta-analytic study concerning the effectiveness of information and communication technologies (ICT) interventions for IPV victims revealed that they are mainly effective in the screening, disclosure, and prevention of IPV [24]. The use of ICT can be helpful if victims consequently use formal services (such as shelters, civil protection, and legal advocacy services) or turn for support to formal institutions (social services, police, etc.). After using such formal support, they are in turn less likely to reexperience IPV [12].

Women who turn to online social support groups for help differ in their experience of violence and the needs they have (type of support sought). Some ask for help directly (clearly expressing their needs), others indirectly (by providing a detailed description of their situation or experience but without clearly asking for help). We wanted to explore whether differences in expressed needs exist between (self-reported) victims or witnesses of violence (victims or witnesses describing violence in their starting post) and others. Do self-reported victims of violence seek different types of support? How do they express their needs? Do they receive more social support than non-victims or non-reported victims? To address these research questions, we retrieved posts from the first online support group for women to appear in Slovenia, Women in Need, and performed content analysis of a randomly selected sample of starting posts.

## 2. Materials and Methods

**Figure 1:**
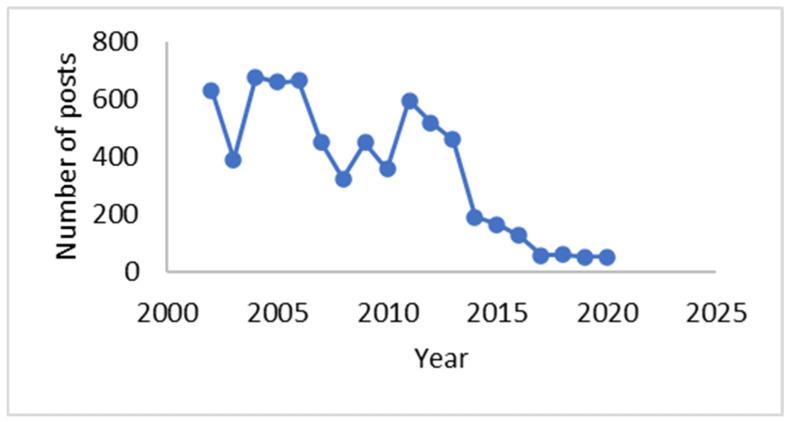
The distribution of the posts in the online support community Women in Need through the years.

The research included retrieval and content analysis of all posts between 2 February 2002 and 20 November 2020 from the earliest online support communities for women to emerge in Slovenia, Women in Need (Ženske v stiski), at the website med.over.net. The community is moderated by social workers and psychologists who work with female victims of IPV violence. An interested person needs a user account to participate in the online discussions. Overall, there were 1455 threads and 6909 posts from 4268 unique users. The data were provided by med.over.net and included information about the thread ID, thread title, username, thread date, post ID, post title, post text, and post date. The online portal was created for the company Styria Media si, d.o.o. The user who submits content to Styria Media si, d.o.o., agrees that by submitting the content to Styria Media si, d.o.o., all its material copyright and related rights are transferred as well. In doing so, the user determines that it is not necessary to state his/her authorship or ownership of related rights when publishing the work.. The distribution of all posts through the years is presented in Figure 1. It is evident that the number of posts has been decreasing since 2012 and that there have been less than 100 posts per year since 2017.

There were 1386 starting posts, among which 600 were randomly selected and manually inspected by two independent coders. The coding included assigning codes regarding the presence of a description of violence in a post, the type of violence, whether the violence was ongoing, what role the post’s author was (victim, witness, perpetrator, other), and who was the perpetrator. In addition, the motives for visiting the online community were identified. These were coded as informational support, emotional support, instrumental support, and emotional release. Results concerning the type of social support users seek online have already been documented elsewhere [19].

The research was approved by the Ethics Commission of the Faculty of Criminal Justice and Security, University of Maribor, Slovenia (ethical approval no. 0208-2023).

The number of times violence was described resulted in a high level of agreement between the coders (Cohen’s kappa equaled 0.80; *p* < 0.001). Interrater agreement in assigning physical violence as inspected by Cohen’s kappa was 0.79 (*p* < 0.001), 0.80 for sexual violence (*p* < 0.001), and 0.82 for psychological violence (*p* < 0.001). The lowest agreement was established for assigning economic violence (κ = 0.37; *p* < 0.001). Following the low level of agreement, this type of violence was not inspected in further analysis. All codes were manually inspected by a third party and all discrepancies were resolved.

The motives for visiting the online support group (OSG) were emotional release and seeking informational, emotional, or instrumental support. Cutrona and Suhr’s definition of types of social support was considered [8]. The agreement between coders, as measured by Cohen’s K, was 0.76 (*p* < 0.001), signifying substantial agreement between coders, and overall, the two coders assigned the same codes to 94% of the posts. All discrepancies were discussed and further examined by a third, independent coder until consensus and a final decision were reached. The coding process is described in greater detail in the article by Erčulj and Pavšič Mrevlje [19].

Categorical variables were described by frequencies and percentages, and continuous ones were described by medians and interquartile ranges (IQR) due to their non-normal distribution. The association between the description of violence and the motive for visiting the OSG was tested by the chi-square test. The association between the type of violence described and the motive for visiting the OSG was inspected by the chi-square test or likelihood ratio test, as appropriate. Overall, 80 starting posts were excluded from this analysis as they did not include support seeking (these posts included offers of (mainly) informational support). The difference in the number of replies received according to the content describing violence was tested by the Mann–Whitney U test. The association between the type of violence described in the post and the motive for visiting the OSG was tested by multiple logistic regression. *p* < 0.05 was treated as statistically significant.

## 3. Results

**Figure 2:**
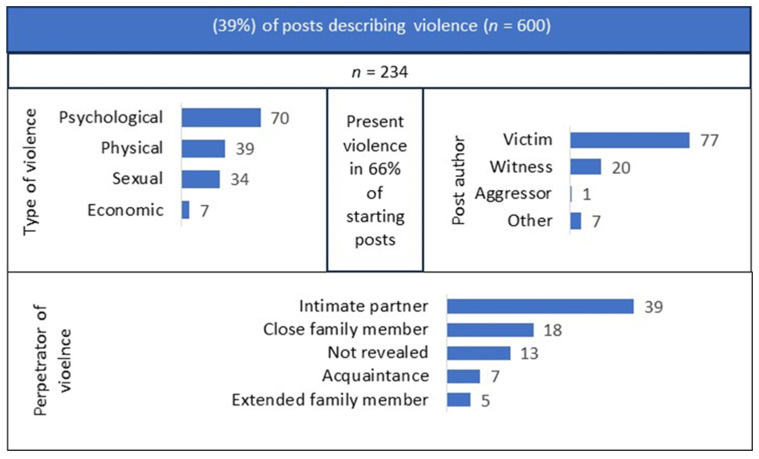
Violence described in the starting posts.

There were 234 (39%; 95% CI: 35–43%) starting posts describing violence, among which 154 (66%; 95% CI: 60–72%) described the present situation (Figure 2). The most frequent type of violence described was psychological (70%; 95% CI: 64–76%), followed by physical (39%; 95% CI: 32–45%), sexual (34%; 95% CI: 28–41%), and economic (7%; 95% CI: 4–10%). A single post could also describe a range of types of violence.

One post describing psychological violence in part states:

“*If the child is a little naughty, he immediately threatens to walk away and then everything will be completely OK. Then the child cries and calls after him but he does not respond. He never says that he was just joking, or in short, he doesn’t know how to comfort either me or the child*.”

An example of physical violence is seen in the following excerpt of a post:

“*We have a problem with our 25-year-old son who doesn’t stick to work, gets drunk all the time, and lives day by day. Because he refused the work offered to him through the social service, he is now deprived of all social rights for half a year. On top of that, he and my husband get into physical fights*.”

Sexual violence is mentioned in the following part of one post:

“*I wonder what I can do if I find out about sexual harassment or abuse? Do I contact the police, social services? And what do they do if they get a report?*”

Economic violence is described in the post below:

“*The boy is completely without energy because he uses it all to survive in the clutches of his parents. He receives money from the social service, but his parents take that money for themselves*.”

An example of a post not describing violence is the following:

“*I have missed a forum where women dare to say what was happening to them. I have missed talking to my peers, even if it’s not in the form of a live meeting. Women, open your hearts, let’s help each other through conversation and professional help*.”

OR

“*I am 12 weeks pregnant and everything seems to indicate that my employment will end prematurely through no fault of my own (otherwise, I have a contract until February 2003). Can this really happen? Do I have any rights as a pregnant woman*?”

Authors of posts were mainly a victim (77%; 95% CI: 72–83%) and/or a witness (20%; 95% CI: 15–26%) of violence. The perpetrator in the situations described was mostly an intimate partner (39%; 95% CI: 32–45%) or a primary family member (18%; 95% CI: 13–23%).

**Figure 3:**
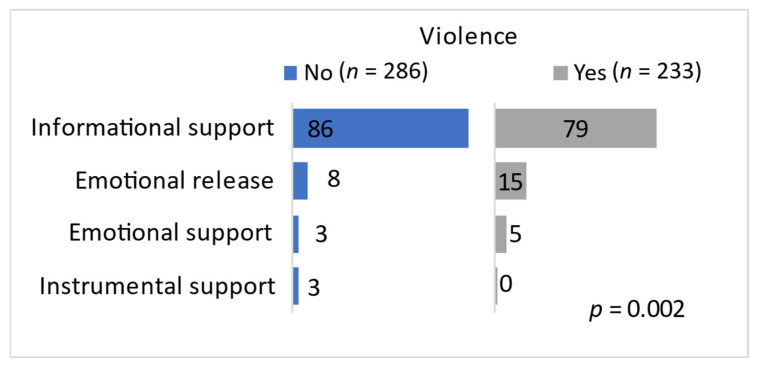
Social support and a description of violence in a post.

Authors of the starting posts were mostly seeking informational support (83%) and emotional release by writing their personal experience (11%), and less frequently looking for emotional (4%) or instrumental (2%) support [19]. Figure 3 illustrates each type of social support sought in posts (not) describing violence. In both instances, users were generally seeking informational support, although the authors of posts describing violence did this less than others (*p* = 0.002). The latter sought emotional release to a bigger extent than the former. The seeking of instrumental support was rare but was always expressed in posts not describing violence.

**Figure 4:**
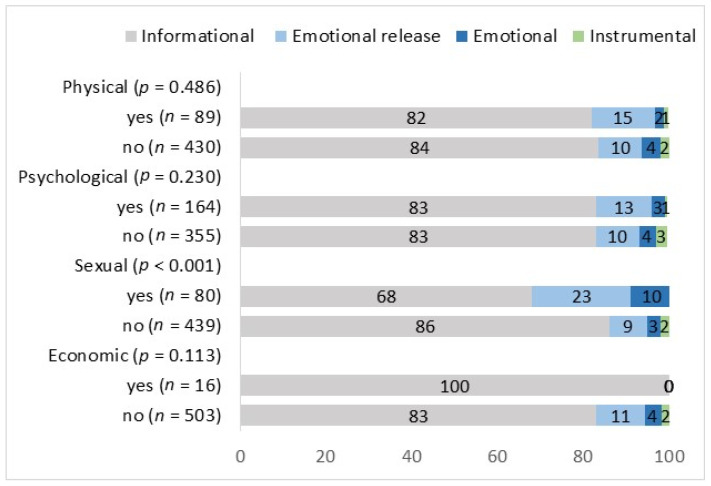
Type of violence described and seeking informational and emotional support.

The difference in seeking each type of support according to the type of violence described in a post was explored and is summarized in Figure 4. The share of participants describing sexual violence who sought informational support was smaller than the share of participants not describing this type of violence, while authors of posts describing economic violence were solely looking for informational support. Only authors describing physical or psychological violence sought instrumental support. The difference in the type of support authors were looking for according to the violence described in the post was statistically significant only between those describing sexual violence in comparison to those who did not (*p* < 0.001). Authors of posts describing sexual violence were to the highest extent (10%) looking for emotional support and emotional release (23%) compared to authors who did not describe this type of violence (3% and 9%, respectively).

The association between each type of violence and seeking informational or emotional support or emotional release was explored by multiple multinomial logistic regression and the results are summarized in Table 1. When controlling for all types of violence described in a starting post, only sexual violence is statistically significantly associated with the type of support sought. Authors of posts describing sexual violence were more likely to seek an emotional release (OR = 3.42 (95% CI: [1.81; 6.49]) and emotional support (OR = 4.77 (95% CI: [1.83; 12.47]) than those not describing sexual violence.

The median (IQR) number of responses to a starting post according to the description and type of violence is shown in Table 2. Posts including the description of violence were followed by a larger number of posts than those not describing violence (*p* < 0.001). This applies to posts describing sexual, physical, and psychological violence, but not posts describing economic violence. At least half the posts describing violence, namely sexual and physical violence, received three or more replies, while at least half of those without violent content received two or more replies.

## 4. Discussion

We analyzed 600 randomly selected starting posts from the first online support community to appear in Slovenia called Women in Need. Our results show that the posts were generally written by victims of violence themselves (77%), with 39% of these posts directly describing the violence they had been experiencing. Two-thirds of these were current violent situations. Psychological violence prevailed, followed by physical and sexual, and then economic.

Congruent with other studies of social support provided online [14,18], the authors of the analyzed starting posts mainly sought informational support (83%), regardless of the post content describing violence. Although (self-reported) victims or witnesses of violence, similar to non-self-reported victims, most commonly visited an online community to gather information to help them solve their situation, the two groups varied in their needs. A clear request for support was more frequently expressed by authors not describing violence, whereas victims (or witnesses) of violence sought an emotional release to a greater extent—by describing their experience—and were not specifically asking for help. In this sense, they are similar to victims of sexual violence who came to an online support group to share their story described by O’Neill [21]. Our tentative interpretation of the observed differences is that the authors of non-violence-describing posts were using a direct strategy for seeking support, including a straightforward request for help, as suggested by Barbee and Cunninham [25]. Such posts likely do not need a description of (violent) situations, but directly request specific information to solve the issue. Further confirmation of this arises from our finding that the need for instrumental support was also only expressed in posts not specifically describing violence.

Seeking some kind of emotional support was the second most common need in our findings (emotional release 11%; emotional support 4%). Going one step further, the multiple multinomial logistic regression revealed that such support was sought significantly more often by authors describing sexual violence than those writing about other types of violence. Similar results were found in a study of rape survivors visiting a public forum on Reddit [21] and in a study of Twitter messages posted by female IPV victims, where sexual abuse is common [20].

What women victims of sexual harassment need first and foremost is legal and psychological support [26]. However, to be able to ask for it, the person must have relative mental clarity about the experience, and the emotional strength to come forward about it. Perhaps the finding that such victims seek emotional support is specifically linked with this kind of violence given that its consequences can be even more severe and overwhelming compared to other types of violence. In fact, rape and sexual molestation are most likely to be associated with post-traumatic stress disorder [25,26]. Therefore, seeking emotional support that includes validation of the victim’s experience and offers clarification and labeling might be their central motivation for posting online at the outset, as was established in a systematic literature review by Gorissen et al. [23]. It might represent the first step to a more coherent understanding of the violent situation/experience that allows a more strategic approach to solving it. Namely, emotion dysregulation, linked to post-traumatic stress and intimate partner violence, may include a lack of awareness, lack of understanding, and difficulty in modulating emotions, among others [27]. In these cases, indirectly asking for help by describing the experience of violence (and similar) might be the only possible way of reaching out at that stage.

Interestingly, posts describing violence received more reactions than those that did not. Perhaps the more direct (informational and instrumental) support seeking in the posts that did not mention experiences of violence call for (one) straightforward and concrete answer. On the other hand, starting posts that include descriptions of violence might trigger different psychological contents more widely in readers, who consequently offer various and numerous responses (i.e., encouragement, sharing similar experiences). It has been demonstrated that self-disclosure, which is greater in posts describing violence, elicits a bigger amount of emotional support [28]. Further, more detailed personal stories function as a basis for self-identification and self-comparison and attract the stronger engagement of users [29]. The finding that posts describing violence elicit more social support is important because previous studies showed that social support can speed up life changes for victims [11], encourage them to leave their abusive partner, and seek face-to-face help [12,13] in formal institutions or shelters. Studies suggest that such help is the most effective [12]. The path to the necessary life changes is especially important for those women who do not have a supportive family, helping them towards recovery. Strong social support from family members tends to eliminate or at least lower the severity and frequency of abuse [10]. Further research is needed with regard to the direct experience of women in such online communities. It would be important to assess how many women who turned to an online community for help then acted by leaving the abusive relationship. A review of online interventions shows that they tend to be most effective in encouraging women to take the first step, but less effective in dealing with the steps that follow [13]. While the remaining steps are therefore better left to the formal support networks, the first step towards recovery is crucial. The key role of online communities is to encourage and lead women to take this first step. The high share of informational support starting posts supports this conclusion. Moreover, previous content analysis of all the posts in this online support community [19] revealed that the second largest topic of online discussions is advising and educating women about their possibilities and contacts with shelters, etc., made by moderators—social workers and psychologists—working in safe houses.

## 5. Conclusions

Our study on online posts of women victims of violence reveals that there is an association between the needs expressed and the experience of violence described. The online support group users most frequently sought informational support, but those describing sexual violence more often asked for emotional support than those without mentioning violence in the starting post. Moreover, posts specifically describing violence were more likely to bring support than short posts directly asking for help. Based on the results of our study, we can conclude that online support groups have an important role in educating and encouraging women to take a further step toward a solution to their discouraging situation. Such online support is even more critical when women seeking help do not have strong social ties. Victims or witnesses of violence seem to ask for help more indirectly by describing the violent situation. Such a search for help evokes a higher number of supportive reactions among OSG users. At this stage, it is paramount that victims receive encouragement and clear instructions about the steps they can take to improve the situation. Clearly, victims of sexual violence have different needs than victims of other types of violence, and special care must be applied when assisting these victims.

When interpreting the results of our study, some limitations must be mentioned. First, limited resources meant only a sample of the starting posts was examined and annotated. Still, probability sampling, namely, the simple random sampling method, was used to select starting posts, which, by calculating confidence intervals of observed shares, allows for conclusions that are representative of the population [30]. The nature of the research was non-invasive, with the drawback being that only content the participants were willing to share online could be analyzed. No specific questions from the researcher to elaborate on some topics were possible. On the other hand, such a non-invasive research approach could not harm (in the sense of secondary victimization) victims in any way and provided them with the anonymity they wanted to preserve by posting under a pseudonym. The results further indicated that psychological violence is the most common type of violence reported online, which is consistent with findings of more rigorous research approaches and adds credit to our conclusions [5]. Further research to support our conclusions is necessary. Qualitative research, including victims of violence managing to improve their situation, would bring more clarity about the steps these women took to resolve their situation, whether they used online support groups, and if and how these groups were of any help. Adding interviews with the moderators would additionally widen the understanding of the dynamics of seeking online help found in our research.

Our study corroborates the importance of the help, information, and support that is offered online for women victims of violence. However, it also reveals that help-seeking (in person and online) is nuanced. Therefore, specific and more personalized support programs regarding the type of violence should be offered.

## Figures and Tables

**Table 1:** Association between types of violence and seeking informational or emotional support or emotional release (results of multiple logistic regression).

	Emotional Release vs. Informational Support		Emotional Support vs. Informational Support	
	OR (95% CI)	*p*	OR (95% CI)	*p*
Physical	1.08 (0.44; 2.7)	0.862	0.47 (0.08; 2.92)	0.419
Verbal	1.49 (0.68; 3.26)	0.323	1.25 (0.34; 4.56)	0.735
Sexual	3.42 (1.81; 6.49)	<0.001	4.77 (1.83; 12.47)	0.001

**Table 2:** Median (IQR) number of responses to the starting post by the presence and type of violence described in the post (results of the Mann-Whitney U test).

	No	Yes	*p*
Violence	2 (1–3)	3 (1–6)	<0.001
Sexual	2 (1–4)	3 (2–5)	<0.001
Physical	2 (1–4)	3 (1–7)	<0.001
Psychological	2 (1–4)	3 (1–6)	<0.001

## Data Availability

Data can be provided upon request from the authors of the article with permission of the founders of the forum Women in Need. Restrictions apply to the availability of these data. Data was obtained from the founders of the Women in Need forum and are available from the authors with the permission of the founders of the forum. Data can be obtained upon request from the authors upon approval of the med.over.net.
